# Adverse Drug Reactions to Antihypertensive Therapy: A Prospective Evaluation of Causality and Severity Using the World Health Organization-Uppsala Monitoring Centre (WHO-UMC) and Hartwig-Siegel Scales

**DOI:** 10.7759/cureus.105476

**Published:** 2026-03-19

**Authors:** Karan Suneja, Shalini Singh, Sarvesh Singh, Rahul Kumar, Narendra Kumar, Rakesh Dixit, K K Sawlani, Amit Kumar, Satish Kumar, Ambuj Yadav, Deepak Bhagchandani

**Affiliations:** 1 Pharmacology and Therapeutics, King George's Medical University, Lucknow, IND; 2 Internal Medicine, King George's Medical University, Lucknow, IND

**Keywords:** adverse drug reactions, antihypertensive drugs, epidemiology, hartwig-siegel scale, hypertension, pharmacovigilance, who-umc scale

## Abstract

Background: Hypertension is one of the most prevalent chronic non-communicable diseases worldwide and remains a leading cause of cardiovascular morbidity and mortality, particularly in low- and middle-income countries such as India. Although antihypertensive pharmacotherapy is effective in reducing cardiovascular risk, adverse drug reactions (ADRs) may compromise treatment adherence and long-term blood pressure control. Systematic identification and evaluation of ADRs using validated causality and severity assessment tools are essential to strengthen pharmacovigilance and optimize therapeutic outcomes. This study is a continuation of our previously published work evaluating prescribing trends of antihypertensive medications in the same tertiary care setting and study population. The present study focuses on the incidence, pattern, causality, and severity of ADRs associated with antihypertensive therapy.

Methods: A prospective observational study was conducted over one year from February 15, 2025, to February 15, 2026, in the Medicine Outpatient Department of a tertiary care teaching hospital in India. Adult patients newly diagnosed with hypertension, defined as systolic blood pressure ≥130 mmHg and/or diastolic blood pressure ≥80 mmHg, documented on at least two separate visits and initiated on antihypertensive therapy, were enrolled. Blood pressure measurements were obtained using a validated sphygmomanometer following standard protocol. Patients were followed longitudinally after treatment initiation, and suspected ADRs were documented using structured case record forms. Causality was assessed using the World Health Organization-Uppsala Monitoring Centre (WHO-UMC) scale, and severity was graded using the Hartwig-Siegel severity assessment scale. Descriptive statistical analysis was performed using the IBM SPSS Statistics for Windows, version 24 (released 2016; IBM Corp., Armonk, New York, United States).

Results: A total of 250 newly diagnosed hypertensive patients were included in the study. The majority of patients were aged > 50 years (150, 60%), overweight or obese (175, 70%), literate (245, 98%), and belonging to socioeconomic Class III (242, 96.8%). Diabetes mellitus (119, 47.6%) and cardiovascular disease (57, 22.8%) were the most common comorbidities. During follow-up, 31 ADRs occurred, corresponding to an incidence of 31 (12.4%). Gastrointestinal and central nervous system reactions were most frequent (six, 19.4% each), followed by cardiovascular (five, 16.1%) and musculoskeletal reactions (four, 12.9%). Angiotensin receptor blockers, particularly telmisartan, were most commonly implicated. According to the WHO-UMC, 14 (45.2%) ADRs were probable and 17 (54.8%) possible. Hartwig-Siegel assessment revealed that most ADRs were mild (27, 87.1%), three (9.7%) moderate, and one (3.2%) moderate-to-severe; no ADR fulfilled WHO seriousness criteria. Most of them improved after dose adjustment or drug discontinuation.

Conclusion: ADRs associated with antihypertensive medications were relatively infrequent and predominantly mild in severity; however, their occurrence highlights the importance of active pharmacovigilance and early detection strategies. Integration of ADR monitoring into routine clinical practice may enhance medication safety, improve adherence, and contribute to better long-term hypertension management outcomes.

## Introduction

Hypertension continues to represent one of the most significant global public health challenges and is a leading cause of cardiovascular morbidity, functional disability, and premature mortality worldwide [1,2]. The global burden of hypertension is steadily increasing, with a disproportionate impact observed in low- and middle-income countries (LMICs), where limitations in population screening, access to healthcare services, and long-term follow-up contribute to inadequate blood pressure control [3,4]. Although effective antihypertensive therapies are widely available, sustained blood pressure regulation remains suboptimal in a substantial proportion of patients, largely due to poor treatment adherence [5]. Adverse drug reactions (ADRs) constitute a major factor contributing to non-adherence and premature discontinuation of therapy. Evidence from tertiary and primary healthcare settings suggests that approximately 16-40% of patients experience ADRs related to antihypertensive medications, with commonly reported symptoms including dizziness, polyuria, cough, peripheral edema, and gastrointestinal disturbances [6,7]. A large tertiary-care study conducted in Nigeria reported that 18.1% of patients with hypertension experienced ADRs, and nearly half of them required treatment modification or discontinuation, highlighting the clinical relevance of drug intolerance [8]. Furthermore, the likelihood of ADR occurrence increases with the number of prescribed medications, emphasizing the contribution of polypharmacy to adverse outcomes [9]. Recent research has also demonstrated the substantial impact of ADRs on patient-reported outcomes. A multicenter primary-care study from Indonesia in 2025 reported that patients experiencing ADRs were seven times more likely to exhibit non-adherence and had significantly poorer health-related quality of life compared with those without ADRs. Although most reactions were mild to moderate in severity, they were sufficient to impair daily functioning and compromise treatment persistence, underscoring the importance of early identification and management [10]. In India, the prevalence of hypertension has increased markedly over the past two decades, accompanied by persistent gaps in disease awareness, treatment initiation, and long-term control, particularly between rural and urban populations [11]. A considerable proportion of affected individuals remain undiagnosed or inadequately treated, thereby increasing their risk of cardiovascular complications [12]. These challenges reinforce the importance of rational prescribing practices, comprehensive patient education, and close therapeutic monitoring. Contemporary guideline-based management frameworks, particularly the updated American College of Cardiology/American Heart Association (ACC/AHA) recommendations, emphasize individualized drug selection based on patient age, comorbidities, and overall cardiovascular risk, along with early treatment initiation and sustained adherence [13,14]. However, real-world prescribing patterns frequently deviate from guideline recommendations due to factors such as drug availability, clinician preferences, patient comorbidity profiles, and tolerability concerns, with ADRs being a major determinant of treatment modification and discontinuation [8,10]. Pharmacovigilance systems play a central role in the detection, evaluation, and prevention of medication-related harm. The World Health Organization (WHO) advocates structured ADR monitoring through standardized reporting mechanisms and international data sharing via the Programme for International Drug Monitoring, which facilitates early signal detection using large global safety databases. Standardized assessment tools, such as the WHO-Uppsala Monitoring Centre (UMC) causality scale, are recommended for consistent attribution of suspected ADRs, whereas severity grading instruments, including the Hartwig scale, support clinical decision-making, prioritization of interventions, and optimal allocation of healthcare resources [15]. In addition to severity assessment, classification of ADR seriousness is an important pharmacovigilance parameter, particularly in national reporting systems, such as the Pharmacovigilance Programme of India (PvPI). Serious ADRs - defined as events resulting in death, life-threatening conditions, hospitalization, disability, congenital anomalies, or other medically important conditions - are prioritized for regulatory monitoring and safety signal detection. Therefore, evaluation of ADRs using causality, severity, and seriousness criteria provides a more comprehensive assessment of drug safety in clinical practice [15]. In India, pharmacovigilance activities under national programs are expanding; however, underreporting, variable clinician engagement, and inconsistent documentation - particularly in outpatient settings - continue to limit the effectiveness of safety surveillance. These limitations highlight the need for institution-based monitoring systems, active reporting strategies, and integration of pharmacovigilance into routine clinical practice to strengthen medication safety and improve patient outcomes [15]. This study is a continuation of our previously published work evaluating prescribing patterns of antihypertensive medications in the same tertiary care setting and study population. Building upon those findings, the present research focuses on the safety profile of antihypertensive therapy. Therefore, the present study was undertaken to evaluate the pattern, causality, and severity of ADRs associated with antihypertensive medications using the WHO-UMC causality criteria and the Hartwig severity assessment scale in a tertiary care teaching hospital in India.

## Materials and methods

A prospective observational study was conducted over one year from February 15, 2025, to February 15, 2026, in the Medicine Outpatient Department of King George’s Medical University, Lucknow, India. Sample size was estimated using the WHO prevalence formula in accordance with standard methodological recommendations. Based on a previously reported ADR prevalence of 30.71% [16], the required sample size was calculated, and 250 patients were enrolled in the study. Adult patients aged 18 years and above with newly diagnosed essential hypertension who were initiated on at least one oral antihypertensive medication were eligible for inclusion. Newly diagnosed hypertension was defined in accordance with standard clinical criteria. Patients with pregnancy-induced hypertension, hypertensive emergencies, secondary hypertension due to renal or hepatic disorders, substance abuse, impaired decision-making capacity, or those unwilling to provide informed consent were excluded from the study. All enrolled participants were followed for six months after initiation of antihypertensive therapy. During scheduled follow-up visits and routine outpatient consultations, suspected ADRs were actively identified and documented using structured case record forms. In addition, patients were encouraged to report any adverse symptoms experienced during treatment. Each reported ADR was evaluated for causality using the WHO-UMC causality assessment scale [17]. According to this standardized method, ADRs were categorized as certain, probable/likely, possible, unlikely, conditional/unclassified, or unassessable/unclassifiable based on the temporal relationship with drug administration, response to dechallenge, and the presence of alternative explanations. Severity assessment was performed using the Hartwig-Siegel scale [18], which classifies reactions as mild (Levels 1-2), moderate (Level 3), or moderate to severe (Level 4), based on clinical impact and required interventions. All collected data were compiled and analyzed using the IBM SPSS Statistics software, version 24.0 (released 2016; IBM Corp., Armonk, New York, United States). Descriptive statistical methods were employed to summarize demographic characteristics, clinical variables, and ADR profiles. Continuous variables are expressed as means and standard deviations, whereas categorical variables are presented as frequencies and percentages. The Chi-square test was used to evaluate associations between categorical variables. Bivariate logistic regression analysis was performed to identify factors associated with the occurrence of ADRs, and the results are expressed as odds ratios (ORs) with 95% confidence intervals (CIs). A p-value < 0.05 was considered statistically significant.

## Results

Participant characteristics

During the study period, 268 patients were screened for eligibility. Of these, 250 patients fulfilled the inclusion criteria and were enrolled in the study. Eighteen patients were excluded from the final analysis, of whom 12 patients did not return for follow-up visits, four patients were referred to other healthcare facilities, and two patients withdrew consent, citing personal reasons. Thus, 250 patients with newly diagnosed hypertension were included in the final analysis and were followed for six months. The demographic profile revealed that hypertension was predominantly observed among older adults, with the majority of participants aged above 50 years. Most patients were overweight or obese, and a large proportion were male, married, and literate. The majority belonged to socioeconomic Class III, based on family income, occupation, and per capita income. Overall, the demographic distribution represents a typical hypertensive outpatient population attending a tertiary care hospital in India. The baseline sociodemographic and clinical characteristics of study participants are summarized in Table 1. 

**Table 1:** Sociodemographic characteristics of study participants SD: standard deviation

Parameter	Category	n (%)/mean ± SD
Age (years)	Mean ± SD	54.93 ± 13.01
Sex	Male	143 (57.2)
BMI (kg/m²)	Mean ± SD	27.81 ± 5.95
BMI category	Overweight/obese	175 (70.0)
Marital status	Married	244 (97.6)
Education	Literate	245 (98.0)
Socioeconomic class	Class III	242 (96.8)
Substance use	Tobacco/alcohol use	41 (16.4)
Comorbidities	Diabetes mellitus	119 (47.6)
	Cardiovascular disease	57 (22.8)

Present medical condition and comorbidities

Assessment of baseline medical conditions revealed that a substantial proportion of patients had associated comorbid illnesses depicted in Table 1 at the time of hypertension diagnosis. Diabetes mellitus was the most prevalent comorbidity, followed by pre-existing cardiovascular disease, indicating a high burden of metabolic and cardiovascular risk among patients with newly diagnosed hypertension. These comorbid conditions necessitated careful selection and monitoring of antihypertensive therapy during follow-up.

Drugs used in the study population during hypertension treatment

**Figure 1:**
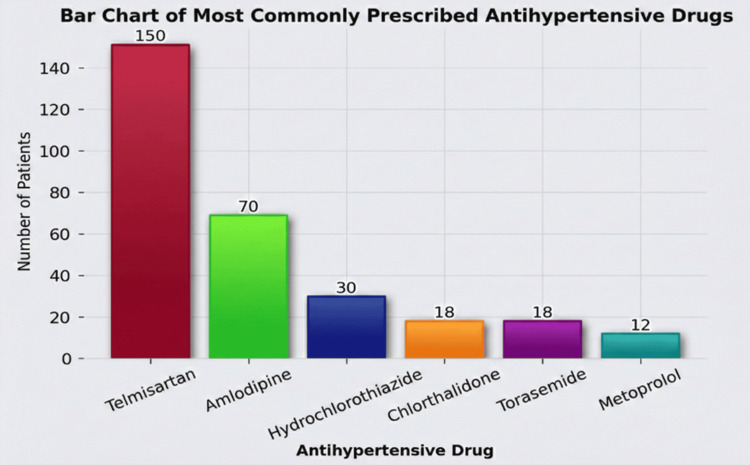
Distribution of commonly prescribed antihypertensive drugs in the study population

All enrolled patients were initiated on antihypertensive therapy for the first time during the study period. Treatment regimens were individualized based on blood pressure levels, associated comorbidities, and the clinical judgment of the treating physician. Combination therapy was prescribed more frequently than monotherapy, reflecting the need for multi-drug regimens in patients with higher blood pressure levels or associated cardiovascular risk factors. Among the antihypertensive agents prescribed, telmisartan was the most commonly used drug, either as monotherapy or in combination with other agents. Other frequently prescribed drugs included amlodipine and cilnidipine, whereas diuretics, such as hydrochlorothiazide and chlorthalidone, were also commonly used. A bar chart depicting the distribution of the commonly prescribed antihypertensive drugs in the study population is shown in Figure 1.

Associated ADRs during antihypertensive treatment

During the six-month follow-up period, 31 ADRs were documented among the study participants, corresponding to an overall incidence of 31 (12.4%). For standardized reporting and international comparability, all ADRs were classified according to the Medical Dictionary for Regulatory Activities (MedDRA) at the System Organ Class (SOC) level. As shown in Table 2, nervous system and gastrointestinal disorders were the most frequently affected categories, each accounting for six (19.4%) of the reported reactions. These were followed by cardiac disorders (five, 16.1%), general disorders and administration site conditions (four, 12.9%), and musculoskeletal disorders (four, 12.9%). Respiratory, renal, and dermatological reactions were observed less frequently. The clinical manifestations and implicated antihypertensive drug classes associated with these ADRs are summarized in Table 3. Neurological manifestations mainly consisted of headache and dizziness, reflecting central nervous system effects related to blood pressure reduction and vasodilatory therapy. Gastrointestinal reactions, including abdominal discomfort, diarrhea, and constipation, were commonly associated with diuretics, angiotensin receptor blockers, and calcium channel blockers. Cardiovascular ADRs, such as bradycardia, palpitations, and hypotension, were consistent with the pharmacodynamic effects of beta-blockers, calcium channel blockers, and diuretics. General symptoms, including nonspecific discomfort, and musculoskeletal complaints, such as muscle cramps, fatigue, and weakness, were also reported and may be attributable to electrolyte imbalance and altered peripheral circulation. Respiratory reactions, including breathlessness and dry cough, were predominantly linked to vasodilatory agents and angiotensin-converting enzyme inhibitors. Renal, urinary, and cutaneous reactions were uncommon and were generally mild in severity. Overall, the observed pattern of ADRs corresponded with established pharmacological profiles of commonly prescribed antihypertensive agents, indicating predictable and mechanistically plausible adverse effects.

**Table 2:** Distribution of ADRs according to MedDRA system organ class (SOC) MedDRA: Medical Dictionary for Regulatory Activities; ADRs: adverse drug reactions

MedDRA SOC	n (%)
Nervous system disorders	6 (19.4)
Gastrointestinal disorders	6 (19.4)
Cardiac disorders	5 (16.1)
General disorders	4 (12.9)
Musculoskeletal disorders	4 (12.9)
Respiratory disorders	3 (9.7)
Renal and urinary disorders	2 (6.5)
Skin disorders	1 (3.2)
Total	31 (100)

**Table 3:** System-wise distribution of adverse drug reactions and implicated antihypertensive drug classes

System	Adverse drug reaction	n (%)	Implicated antihypertensive class
Gastrointestinal	Abdominal pain	2 (6.5)	Calcium channel blocker (CCB)
Gastrointestinal	Diarrhea	3 (9.7)	Angiotensin receptor blocker (ARB)
Gastrointestinal	Constipation	1 (3.2)	Diuretics
Nervous system	Headache	4 (12.9)	CCB
Nervous system	Dizziness	2 (6.5)	ARB
Cardiovascular	Bradycardia	2 (6.5)	Beta-blocker
Cardiovascular	Palpitations	2 (6.5)	CCB
Cardiovascular	Hypotension	1 (3.2)	Diuretics
General disorders	Discomfort	4 (12.9)	ARB
Musculoskeletal	Muscle cramps	2 (6.5)	Diuretics
Musculoskeletal	Fatigue	1 (3.2)	ARB
Musculoskeletal	General weakness	1 (3.2)	CCB
Respiratory	Breathlessness	2 (6.5)	ARB
Respiratory	Dry cough	1 (3.2)	Angiotensin-converting enzyme inhibitor (ACE inhibitor)
Genitourinary	Polyuria	1 (3.2)	Diuretics
Genitourinary	Increased frequency of micturition	1 (3.2)	CCB
Skin	Itching	1 (3.2)	ARB

Causality, severity, drug implication, and clinical outcome of ADRs

Among the 250 newly diagnosed hypertensive patients followed for six months, ADRs were documented in 31 individuals, with each affected patient reporting a single reaction. An overview of ADR characteristics, including causality, severity, management strategies, and clinical outcomes, is presented in Table 4. Causality assessment using the WHO-UMC criteria categorized all reported ADRs as either probable or possible, indicating a consistent temporal relationship between antihypertensive therapy and symptom onset. According to the Hartwig-Siegel severity scale, most reactions were mild and required minimal intervention, whereas a smaller proportion were classified as moderate or moderate-severe, and no life-threatening events were observed. Table 4 summarizes the overall causality assessment, severity grading, and clinical outcomes of ADRs, whereas the specific antihypertensive drugs implicated in individual ADR events are presented separately in Table 5. The distribution of suspected drugs implicated in ADRs broadly reflects the prescribing pattern of antihypertensive drugs observed in the study population, as illustrated in Figure 1. In addition, the potential influence of polypharmacy and concomitant medications as confounding factors was considered during ADR assessment. A proportion of patients were receiving combination antihypertensive therapy, reflecting contemporary clinical practice for achieving adequate blood pressure control. In such cases, attribution of a specific drug to an observed ADR was based on the temporal relationship between drug initiation and symptom onset, known pharmacological profiles of suspected agents, and response to dechallenge where applicable. The possibility of confounding effects from concomitant medications prescribed for comorbid conditions, such as diabetes mellitus and cardiovascular disease, was also evaluated during causality assessment. However, based on WHO-UMC criteria and clinical evaluation, most ADRs were considered pharmacologically plausible and were attributed to the suspected antihypertensive agents. Angiotensin receptor blockers, particularly telmisartan, were most frequently implicated, followed by calcium channel blockers, such as cilnidipine and amlodipine, and diuretics. This distribution largely reflected the prescribing patterns observed in the study population. Other drug classes, including beta-blockers, angiotensin-converting enzyme inhibitors, and adjunctive agents, were associated with fewer reactions. In most cases, ADRs were effectively managed through dose adjustment or modification of therapy. Drug discontinuation or switching was reserved for reactions associated with greater clinical impact or poor tolerability. Many reactions resolved without the need for additional treatment, whereas others required symptomatic or specific therapy. Dechallenge was performed in most patients and resulted in clinical improvement or complete recovery, supporting a causal association, whereas rechallenge was not attempted due to ethical considerations and the availability of alternative treatment options. Overall, clinical outcomes were favorable, with nearly all patients demonstrating complete recovery.

**Table 4:** Profile of adverse drug reactions and causality assessment ADRs: adverse drug reactions, WHO-UMC: World Health Organization-Uppsala Monitoring Centre

Parameter	Category	n (%)
Patients with ADRs	Out of 250 patients	31 (12.4)
Number of ADRs per patient	One ADR	31 (100)
	Two or more ADRs	0 (0)
WHO-UMC causality assessment	Certain	0 (0)
	Probable	14 (45.2)
	Possible	17 (54.8)
	Unlikely	0 (0)
Hartwig-Siegel severity	Mild (Level 1-2)	27 (87.1)
	Moderate (Level 3)	3 (9.7)
	Moderate-severe (Level 4)	1 (3.2)
	Severe (Level 5-7)	0 (0)
Fate of suspected drug	Dose changed	20 (64.5)
	Drug stopped	4 (12.9)
	Medication switched	5 (16.1)
	Frequency altered	2 (6.5)
Treatment for ADRs	No treatment required	12 (38.7)
	Symptomatic treatment	14 (45.2)
	Specific treatment	5 (16.1)
Dechallenge/rechallenge	Dechallenge done	21 (67.7)
	Rechallenge done	0 (0)
	No challenge	10 (32.3)
Outcome	Recovered	30 (96.8)
	Outcome unknown	1 (3.2)

**Table 5:** Drugs implicated in the causation of ADR ADR: adverse drug reaction

Suspected drug	n (%)
Telmisartan	18 (58.1)
Cilnidipine	10 (32.3)
Amlodipine	6 (19.4)
Hydrochlorothiazide	4 (12.9)
Chlorthalidone	4 (12.9)
Ramipril	2 (6.5)
Diltiazem	2 (6.5)
Torasemide	2 (6.5)
Metoprolol	1 (3.2)
Nebivolol	1 (3.2)
Bisoprolol	1 (3.2)
Prazosin	1 (3.2)
Benidipine	1 (3.2)

Risk factors for ADRs in the study population

Bivariate logistic regression analysis was performed to identify factors associated with the occurrence of ADRs among patients receiving antihypertensive therapy. The contingency data used for calculating the ORs are presented in Table 6. Patients aged > 50 years had a significantly higher likelihood of developing ADRs compared with younger patients (OR = 3.13; 95% CI = 1.23-7.95; p = 0.033). Similarly, the presence of diabetes mellitus was significantly associated with an increased risk of ADR occurrence (OR = 2.20; 95% CI = 1.02-4.74; p = 0.045). No statistically significant association was observed between ADR occurrence and patients' sex (OR = 0.89, p = 0.49). Similarly, although patients receiving combination antihypertensive therapy demonstrated slightly higher odds of developing ADRs compared with those receiving monotherapy (OR = 1.06, 95% CI = 0.48-2.33), this association did not reach statistical significance (p = 0.89). Overall, advanced age and the presence of diabetes mellitus emerged as significant predictors of ADR occurrence, whereas sex and type of antihypertensive therapy were not significantly associated with ADR development in the study population.

**Table 6:** Bivariate analysis of adverse treatment outcomes with study variables p < 0.05 is considered statistically significant. OR: odds ratio, CI: confidence interval, ADR: adverse drug reaction

Variable	ADR n (%) (n = 31)	No ADR n (%) (n = 219)	OR	95% CI	χ²	p-value
Age > 50 years	24 (77.4)	126 (57.5)	3.13	1.23-7.95	4.52	0.033*
Male sex	17 (54.8)	126 (57.5)	0.89	0.42-1.89	0.47	0.49
Diabetes mellitus	20 (64.5)	99 (45.2)	2.20	1.02-4.74	4.03	0.045*
Combination therapy	18 (58.1)	123 (56.2)	1.06	0.48-2.33	0.02	0.89
*Significant						

## Discussion

The present study provides real-world insights into ADRs occurring during the early phase of antihypertensive therapy among newly diagnosed patients. The observed ADR incidence of 12.4% is consistent with reports from Nepal, Odisha, and other South Asian regions, where rates ranging from 8% to 20% have been documented, highlighting the continued clinical relevance of ADRs during treatment initiation [19-21].

Neurological and gastrointestinal systems were the most frequently affected organ systems in the present study, in agreement with previous pharmacovigilance studies involving cardiovascular medications [22]. Frequently reported symptoms, such as dizziness, headache, weakness, diarrhea, constipation, and abdominal discomfort, have been widely described across multiple antihypertensive drug classes, including diuretics, angiotensin-converting enzyme inhibitors, and fixed-dose combinations [19,23]. Cardiovascular manifestations, such as bradycardia, palpitations, and transient hypotension, reflected expected pharmacodynamic effects of β-blockers, calcium channel blockers, and combination therapy. Respiratory ADRs, particularly angiotensin-converting enzyme inhibitor-induced cough, were also observed and are well supported by existing evidence [23]. Although less frequent, genitourinary and musculoskeletal complaints contributed to patient discomfort and may negatively influence treatment adherence, emphasizing the need for anticipatory counseling and patient education.

The predominance of neurological, gastrointestinal, and cardiovascular adverse reactions observed in this study can be largely explained by the pharmacological actions and hemodynamic effects of commonly prescribed antihypertensive agents. Symptoms such as dizziness and headache are frequently related to rapid blood pressure reduction, impaired cerebral autoregulation, and vasodilatory effects, particularly during treatment initiation and dose escalation. Gastrointestinal disturbances may result from alterations in electrolyte balance, intestinal motility, and splanchnic blood flow, especially with diuretics and renin-angiotensin system inhibitors. Cardiovascular manifestations, such as bradycardia and hypotension, are primarily attributable to excessive sympathetic inhibition and negative chronotropic effects associated with beta-blockers and certain calcium channel blockers.

Hospitalization due to symptomatic bradycardia in one patient underscores the importance of individualized dose titration and careful clinical monitoring, particularly during the early phases of antihypertensive therapy. Early identification of drug-related adverse effects and timely dose adjustment can help prevent clinically significant complications and improve patient safety during treatment initiation.

An important finding of the present study was the significantly higher likelihood of ADR occurrence among patients aged above 50 years. Patients in this age group demonstrated an increased risk of developing ADRs compared with younger individuals (OR = 3.13; 95% CI = 1.23-7.95; p = 0.016). This increased susceptibility may be attributed to age-related physiological changes, altered pharmacokinetics and pharmacodynamics, reduced renal and hepatic drug clearance, and the presence of multiple comorbid conditions. Furthermore, older patients are more likely to receive multiple medications, which may increase the risk of drug interactions and adverse effects.

The higher susceptibility observed among older patients and individuals with diabetes may also reflect increased comorbidity burden and the potential contribution of polypharmacy. Most of these reactions are potentially preventable through careful dose titration, appropriate drug selection, avoidance of unnecessary drug combinations, regular clinical and biochemical monitoring, and early patient education regarding expected adverse effects and warning signs. Strengthening multidisciplinary collaboration among physicians, pharmacists, and nursing staff may further facilitate early detection and timely management of ADRs in routine clinical practice.

Causality assessment using the WHO-UMC scale categorized all reported reactions as possible or probable, which is typical for observational studies in which rechallenge is not ethically feasible [20]. Previous Indian studies have also demonstrated only moderate agreement between WHO-UMC and algorithm-based tools, such as the Naranjo scale, reflecting inherent methodological limitations in causality assessment under routine clinical conditions.

Severity grading using the Hartwig-Seigel scale indicated that most ADRs were mild and self-limiting, requiring minimal intervention, consistent with findings from other Indian and international cohorts [22-24,19-21]. Given that ADRs represent a major contributor to non-adherence [25] and that uncontrolled hypertension substantially increases cardiovascular morbidity and mortality [1,2,6], early recognition and proactive management of adverse effects are essential to prevent unnecessary treatment discontinuation.

A substantial proportion of ADRs, particularly dose-related and pharmacologically predictable reactions, are potentially preventable. Preventive strategies include rational drug selection based on patient characteristics, gradual dose escalation, regular monitoring of biochemical and clinical parameters, avoidance of unnecessary polypharmacy, and structured patient education regarding early warning symptoms and appropriate reporting pathways [26]. Periodic medication reviews, especially in elderly patients and individuals with multiple comorbidities, can further reduce avoidable drug-related harm.

Contemporary pharmacovigilance frameworks emphasize the integration of ADR monitoring into routine clinical practice through standardized reporting systems, electronic health record-based surveillance tools, and continuous training of healthcare professionals [27]. Large international safety databases, including those maintained under the WHO Programme for International Drug Monitoring, facilitate early signal detection and enable the timely dissemination of safety information to clinicians and regulatory authorities [28]. Strengthening institutional reporting mechanisms and encouraging both clinician- and patient-initiated reporting remain crucial in addressing persistent under-reporting challenges in India [23,24].

Recent advances in digital health and artificial intelligence (AI) offer promising opportunities to further enhance ADR prevention and surveillance. Machine learning models and natural language processing techniques have demonstrated the ability to predict ADR risk, identify safety signals from large datasets, and analyze unstructured clinical narratives and patient-reported outcomes [29]. Integration of AI-driven pharmacovigilance tools with clinical decision support systems may enable personalized risk stratification, optimized dosing strategies, and early identification of high-risk patients. In addition, mobile health applications and electronic reporting platforms can facilitate real-time patient engagement, improve follow-up compliance, and support timely documentation and management of adverse events.

Despite its strengths, including a real-world design and structured assessment using validated tools, this study has several limitations. The single-center setting, modest sample size, relatively short follow-up duration, and reliance on patient self-reporting may have resulted in underestimation of delayed or subclinical reactions. Consequently, the generalizability of these findings to broader and more diverse populations may be limited. Larger multicentric studies with longer follow-up periods are warranted to further validate these observations.

Overall, the largely predictable and manageable ADR profile observed in this study supports current antihypertensive prescribing practices while reinforcing the importance of individualized therapy, anticipatory counseling, early follow-up, and sustained pharmacovigilance. Combining conventional surveillance systems with emerging digital and AI-based tools may further strengthen medication safety and contribute to improved long-term outcomes in patients with hypertension.

## Conclusions

This prospective observational study provides real-world evidence on the incidence, pattern, causality, and severity of ADRs associated with antihypertensive therapy in newly diagnosed patients at a tertiary care hospital in India. The overall incidence of ADRs was modest, and most reactions were mild, predictable, and reversible, indicating that commonly prescribed antihypertensive agents are generally well tolerated when appropriately selected and monitored. However, the higher risk observed among older patients and individuals with comorbid diabetes mellitus highlights the importance of individualized drug selection, cautious dose titration, and close clinical follow-up during treatment initiation. Early recognition and proactive management of adverse effects are essential to prevent avoidable treatment discontinuation and improve long-term adherence and blood pressure control. Strengthening routine pharmacovigilance practices, enhancing patient counseling, and incorporating digital health and AI-based tools for ADR surveillance and risk prediction may further optimize medication safety and therapeutic outcomes in hypertensive populations.

## References

[1] Belay DG, Fekadu Wolde H, Molla MD (2022). Prevalence and associated factors of hypertension among adult patients attending the outpatient department at the primary hospitals of Wolkait tegedie zone, Northwest Ethiopia. Front Neurol.

[2] (2026). A global brief on hypertension: silent killer, global public health crisis: World Health Day 2013. https://www.who.int/publications/i/item/a-global-brief-on-hypertension-silent-killer-global-public-health-crisis-world-health-day-2013.

[3] Kumma WP, Lindtjørn B, Loha E (2021). Prevalence of hypertension, and related factors among adults in Wolaita, southern Ethiopia: a community-based cross-sectional study. PLoS One.

[4] Kearney PM, Whelton M, Reynolds K, Muntner P, Whelton PK, He J (2005). Global burden of hypertension: analysis of worldwide data. Lancet.

[5] Peng X, Wan L, Yu B, Zhang J (2025). The link between adherence to antihypertensive medications and mortality rates in patients with hypertension: a systematic review and meta-analysis of cohort studies. BMC Cardiovasc Disord.

[6] Srinath Reddy K, Shah B, Varghese C, Ramadoss A (2005). Responding to the threat of chronic diseases in India. Lancet.

[7] Ramakrishnan S, Zachariah G, Gupta K (2019). Prevalence of hypertension among Indian adults: results from the Great India Blood Pressure Survey. Indian Heart J.

[8] Olowofela AO, Isah AO (2017). A profile of adverse effects of antihypertensive medicines in a tertiary care clinic in Nigeria. Ann Afr Med.

[9] Rajadhyaksha GC, Reddy H, Singh AK, Oomman A, Adhyapak SM (2023). The Indian registry on current patient profiles & treatment trends in hypertension (RECORD): one year interim analysis. Indian J Med Res.

[10] Insani WN, Wei L, Abdulah R (2025). Exploring the association of adverse drug reactions with medication adherence and quality of life among hypertensive patients: a cross-sectional study. Int J Clin Pharm.

[11] Whelton PK, Carey RM, Mancia G, Kreutz R, Bundy JD, Williams B (2022). Harmonization of the American College of Cardiology/American Heart Association and European Society of Cardiology/European Society of Hypertension Blood Pressure/Hypertension guidelines: comparisons, reflections, and recommendations. Circulation.

[12] Weber MA, Schiffrin EL, White WB (2014). Clinical practice guidelines for the management of hypertension in the community: a statement by the American Society of Hypertension and the International Society of Hypertension. J Clin Hypertens (Greenwich).

[13] Whelton PK, Carey RM, Aronow WS (2018). 2017 ACC/AHA/AAPA/ABC/ACPM/AGS/APhA/ASH/ASPC/NMA/PCNA Guideline for the prevention, detection, evaluation, and management of high blood pressure in adults: executive summary: a report of the American College of Cardiology/American Heart Association Task Force on Clinical Practice Guidelines. Circulation.

[14] Rao SV, O'Donoghue ML, Ruel M (2025). 2025 ACC/AHA/ACEP/NAEMSP/SCAI Guideline for the management of patients with acute coronary syndromes: a report of the American College of Cardiology/American Heart Association Joint Committee on Clinical Practice Guidelines. Circulation.

[15] Prakash J, Sachdeva R, Shrivastava TP, Jayachandran CV, Sahu A (2021). Adverse event reporting tools and regulatory measures in India through outcome of Pharmacovigilance Programme of India. Indian J Pharmacol.

[16] Roy B, Mohanty S, Prasad A, Pattanayak C, Palit R, Chouhan AS (2019). The study of adverse drug reactions of antihypertensive medicines in essential hypertension patients in Hi-Tech Medical College and Hospital, Bhubaneswar, Odisha, India. Int J Basic Clin Pharmacol.

[17] (2026). The use of the WHO-UMC system for standardised case causality assessment. https://www.who.int/publications/m/item/WHO-causality-assessment.

[18] Hartwig SC, Siegel J, Schneider PJ (1992). Preventability and severity assessment in reporting adverse drug reactions. Am J Hosp Pharm.

[19] Abdelkader NN, Awaisu A, Elewa H, El Hajj MS (2023). Prescribing patterns of antihypertensive medications: a systematic review of literature between 2010 and 2020. Explor Res Clin Soc Pharm.

[20] Arshad V, Samad Z, Das J (2021). Prescribing patterns of antihypertensive medications in low- and middle-income countries: a systematic review. Asia Pac J Public Health.

[21] Yang R, Tang J, Kuang M, Liu H (2023). Analysis of prescription status of antihypertensive drugs in Chinese patients with hypertension based on real-world study. Ann Med.

[22] Degli Esposti E, Sturani A, Di Martino M (2002). Long-term persistence with antihypertensive drugs in new patients. J Hum Hypertens.

[23] Ross SD, Akhras KS, Zhang S, Rozinsky M, Nalysnyk L (2001). Discontinuation of antihypertensive drugs due to adverse events: a systematic review and meta-analysis. Pharmacotherapy.

[24] Khurshid F, Aqil M, Alam MS, Kapur P, Pillai KK (2012). Monitoring of adverse drug reactions associated with antihypertensive medicines at a university teaching hospital in New Delhi. Daru.

[25] Mulchandani R, Kakkar AK (2019). Reporting of adverse drug reactions in India: a review of the current scenario, obstacles and possible solutions. Int J Risk Saf Med.

[26] Litvinova O, Yeung AW, Hammerle FP (2024). Digital technology applications in the management of adverse drug reactions: bibliometric analysis. Pharmaceuticals (Basel).

[27] Algarvio RC, Conceição J, Rodrigues PP, Ribeiro I, Ferreira-da-Silva R (2025). Artificial intelligence in pharmacovigilance: a narrative review and practical experience with an expert-defined Bayesian network tool. Int J Clin Pharm.

[28] Nagar A, Gobburu J, Chakravarty A (2025). Artificial intelligence in pharmacovigilance: advancing drug safety monitoring and regulatory integration. Ther Adv Drug Saf.

[29] Dsouza VS, Leyens L, Kurian JR, Brand A, Brand H (2025). Artificial intelligence (AI) in pharmacovigilance: a systematic review on predicting adverse drug reactions (ADR) in hospitalized patients. Res Social Adm Pharm.

